# Exploring Mental Distress Among Older Workers With Mild Cognitive Impairment By Race And Ethnicity

**DOI:** 10.1093/geroni/igaf122.3765

**Published:** 2025-12-31

**Authors:** Ronica Rooks, Mason Pearce, Joyce Lopis, Paulina Erices-Ocampo

**Affiliations:** University of Colorado Denver, Denver, Colorado, United States; Metropolitan State University of Denver, Denver, Colorado, United States; University of Colorado Denver, Denver, Colorado, United States; University of Colorado Denver, Denver, Colorado, United States

## Abstract

Older Black and Latinx adults experience faster cognitive declines and higher dementia prevalence than older White adults. Meaningful activities, like employment, may help slow cognitive declines among older adults with mild cognitive impairment (MCI), but little is known about whether employment can reduce cognitive declines or racial/ethnic disparities in this population. Our pilot study explored mental distress associated with employment among older adults aged 55+ with MCI and gathered insights from community navigators to assess if and how employment may support cognitive functioning across racial/ethnic groups. We conducted three qualitative interviews with older White adults with MCI and two focus groups with Latinx community navigators (n = 12). Data were analyzed using thematic analysis with Atlas.ti. Older White adults with MCI enjoyed working and felt employment supported cognitive functioning through social engagement and mental activity. But, they reported needing family and coworker support. Stress stemmed from complex job tasks and supervisors’ performance expectations, but workplace accommodations improved their job satisfaction. Racial/ethnic workplace challenges were not reported. Latinx community navigators described cultural biases in cognitive assessments, stigma around cognitive health, and limited discussion of cognitive health in Latinx families. They noted economic constraints and family dynamics influencing older adult women’s caregiving roles and barriers to recognizing and addressing cognitive decline in Latinx communities. These findings highlight the potential of employment to support cognitive functioning in older adults with MCI and cultural and structural barriers for Latinx individuals. Ongoing research will include older African-American and Latinx adults and the nationally-representative Health and Retirement Study.

